# Telemonitoring for patients with inflammatory bowel disease amid the COVID-19 pandemic—A cost-effectiveness analysis

**DOI:** 10.1371/journal.pone.0266464

**Published:** 2022-04-07

**Authors:** Jiaqi Yao, Ginenus Fekadu, Xinchan Jiang, Joyce H. S. You

**Affiliations:** School of Pharmacy, Faculty of Medicine, The Chinese University of Hong Kong, Hong Kong SAR, China; Center for Primary Care and Public Health, SWITZERLAND

## Abstract

**Background and aim:**

COVID-19 pandemic burdens the healthcare systems, causes healthcare avoidance, and might worsen the outcomes of inflammatory bowel disease (IBD) management. We aimed to estimate the impact of pandemic-related avoidance on outpatient IBD management, and the cost-effectiveness of adding telemonitoring during pandemic from the perspective of Hong Kong public healthcare provider.

**Methods:**

The study was performed by a decision-analytic model to estimate the quality-adjusted life-years (QALYs) and cost of care for IBD patients before and during the pandemic, and to compare the cost and QALYs of adding telemonitoring to standard care (SC-TM) versus standard care alone (SC) for IBD patients during the pandemic. The sources of model inputs included publications (retrieved from literature search) and public data. Sensitivity analyses were conducted to examine the robustness of base-case results.

**Results:**

Standard care with pandemic-related avoidance (versus without avoidance) lost 0.0026 QALYs at higher cost (by USD43). The 10,000 Monte Carlo simulations found standard care with pandemic-related avoidance lost QALYs and incurred higher cost in 100% and 96.82% of the time, respectively. Compared with the SC group, the SC-TM group saved 0.0248 QALYs and reduced cost by USD799. Monte Carlo simulations showed the SC-TM group gained higher QALYs at lower cost in 100% of 10,000 simulations.

**Conclusions:**

Standard care for IBD patients during pandemic with healthcare avoidance appears to worsen treatment outcomes at higher cost and lowered QALYs. The addition of telemonitoring to standard care seems to gain higher QALYs and reduce cost, and is therefore a potential cost-effective strategy for IBD management during the pandemic.

## Introduction

Inflammatory bowel disease (IBD), including ulcerative colitis and Crohn’s disease, is a chronic gastrointestinal disorder associated with lifelong morbidity, impaired health-related quality of life and increased disability [1, 2]. The global IBD burden continued to rise in the past three decades between 1990 and 2017, with a prevalence of 0.0896% (89.6 per 100,000 population). Since 1990, the number of global cases had increased by 85% to more than 6.8 million in 2017 [1, 3]. In Hong Kong, the incidence of IBD has raised by 30 times from 0.0001% (0.1 per 100,000 population) in 1985 to 0.0031% (3.1 per 100,000 population) in 2014 [4]. With the growing global burden, IBD has been imposing a substantial impact on healthcare systems [5–7]. The estimated annual healthcare expenditure for IBD in 2015 was 7.2 billion USD, and was one of the most expensive gastrointestinal diseases in the US [8]. Furthermore, the annual direct cost of care incurred to patients with IBD was 3 times higher than non-IBD patients [9].

The outbreak of coronavirus disease 2019 (COVID-19) has inflicted unprecedented challenges on the healthcare systems worldwide since 2020. The periodic surge of COVID-19 cases has caused the healthcare resources and priorities to shift towards the needs of pandemic. The routine medical follow-ups of IBD patients, similar to other chronic diseases, have been paused or interrupted by the shortage of healthcare resources as well as the patients’ anxiety on the risk of COVID-19 associated with healthcare facilities [10, 11]. A recent survey in Hong Kong also found that the general population had reduced outpatient visits during the pandemic, and COVID-19 was identified as a significant factor associated with healthcare service avoidance [12]. The impact of the pandemic on the outcomes of IBD management is yet to be investigated.

Telemonitoring has been considered as the core part of electronic healthcare in the future, and it refers to tracking the patients’ disease progression using the information and communication technologies such as mobile (or wearable) devices, or via telephone and internet reporting [13]. Prior to the pandemic, the telemonitoring programs designed for IBD management had demonstrated improvement of treatment outcomes in clinical studies [14–18]. The risk of hospitalization was found to reduce in IBD patients who received care via telemonitoring in a randomized controlled trial [14]. Despite the speedy launching of multiple COVID-19 vaccines, it is expected to take a few years to bring COVID-19 under control worldwide [19]. The pandemic has fast-forwarded the demand of telemonitoring for IBD management, and the telemonitoring implementation amid the pandemic warrants health economic findings to inform the decision-makers on resource allocation. In the present study, we developed a decision-analytic model to (1) estimate the size of impact on treatment outcomes (measured as quality-adjusted life-year (QALY) and cost) of pandemic-related avoidance/interruption of in-person routine management of IBD patients, and (2) to evaluate the potential cost-effectiveness of adding a telemonitoring program for IBD management during the COVID-19 pandemic.

## Methods

### Model design

A decision-analytic model was developed to simulate the treatment outcomes of a hypothetical cohort of Hong Kong adult IBD patients (with ulcerative colitis or Crohn’s disease) managed at the outpatient setting. The model time horizon was one year, and model output measures included direct medical cost and QALYs. The prevalence of patients with ulcerative colitis (56.8%) and Crohn’s disease (43.2%) among IBD patients was adopted from the epidemiology findings (n = 2,575) of a territory-wide population-based IBD registry in Hong Kong. The median age of patients with ulcerative colitis was 52 years and 56% were male; whilst the median age was 40 years and 65% were male in those with Crohn’s disease. The duration of disease in ulcerative colitis and Crohn’s disease groups were 117 months and 84 months, respectively [20].

Decision-analytic modelling, incorporating the clinical, utility and costs inputs from multiple sources, is a well-accepted tool to assess the health and economic outcomes of health technologies over time [21]. International health economic guidelines of data transferability in decision-analytic modelling consider relative treatment effect and side effect to have high transferability even if derived from clinical trials conducted in a population different from the local population [22]. Disease prevalence and healthcare resource utilization are considered to have low transferability, and local references are recommended sources for epidemiology and cost inputs [23]. The impact of model input uncertainties on the robustness model outputs should be examined by sensitivities analyses.

**Fig 1:**
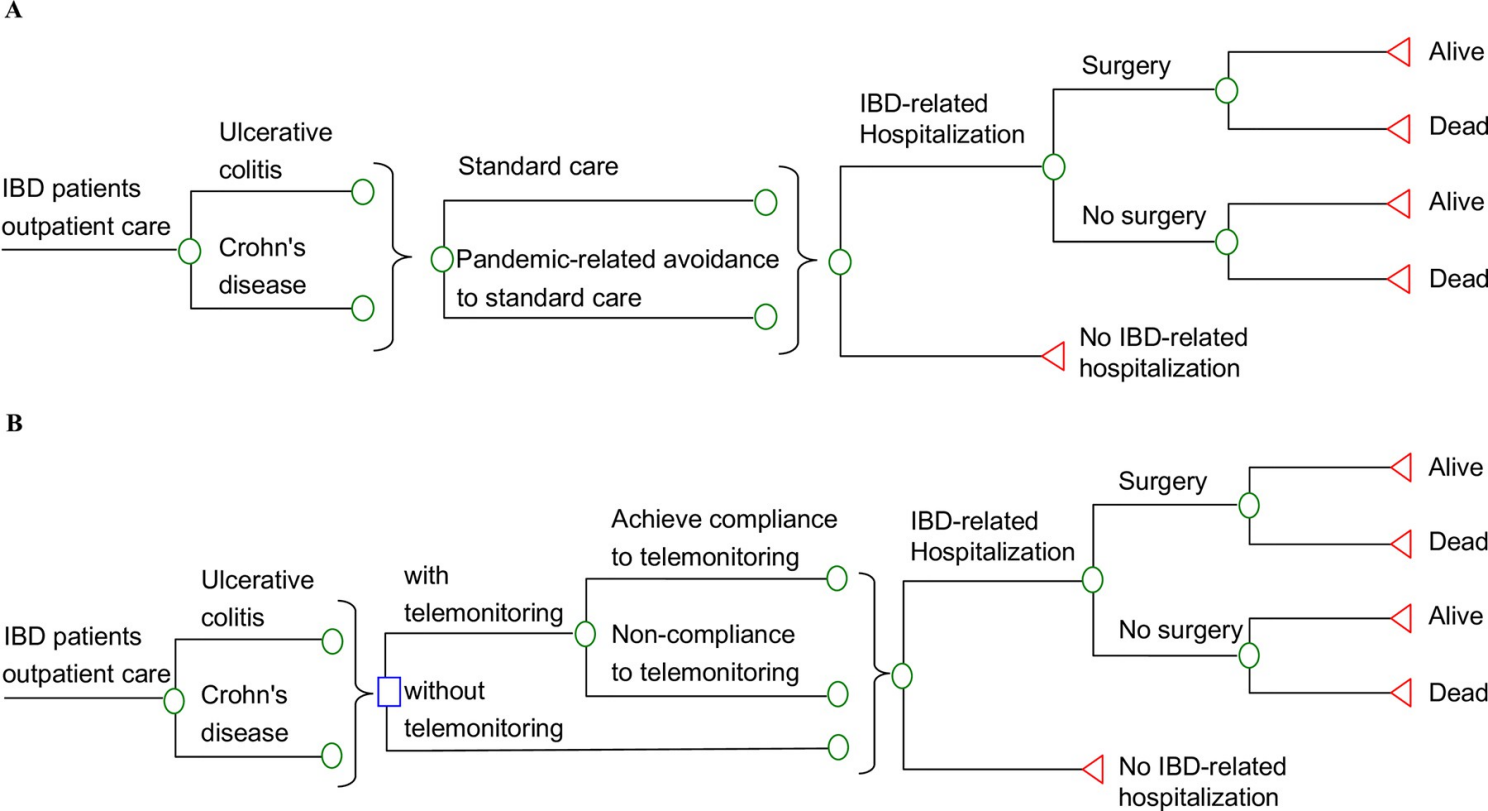
**A.** Simplified decision-analytic models for standard IBD care with and without pandemic-related avoidance. **B.** Simplified decision-analytic models for standard IBD care with and without telemonitoring during the pandemic. IBD, inflammatory bowel disease.

Two model-based analyses were conducted in the present study. The details of model inputs for both model-based analyses are described in subsections “Clinical inputs” and “Cost and utility inputs” below. In analysis 1 (**Fig 1A**), the outcomes of IBD management by standard care before (without healthcare avoidance) and during the COVID-19 pandemic (with healthcare avoidance) were evaluated. IBD patients who received standard outpatient care prior to the pandemic might experience IBD-related hospitalization. The hospitalized patients, with or without surgery, might survive or die. During the pandemic, the patients’ anxiety of the association between COVID-19 and healthcare facilities causes healthcare avoidance and hesitancy to attend routine medical care. The relative change related to healthcare avoidance in standard medical care during the pandemic was therefore included for estimation of the subsequent change in cost and QALYs during the pandemic.

In analysis 2 (**Fig 1B**), two strategies for IBD outpatient management during the pandemic (with healthcare avoidance) were compared: (1) standard care with telemonitoring (SC-TM group) and (2) standard care without telemonitoring (SC group). The standard medical care of adult IBD patients in Hong Kong follows the recommendations for medical management and monitoring of IBD in Asia [24], and was therefore adopted in both analyses 1 and 2 of the present model. In the SC-TM group, the patient received telemonitoring for IBD management in addition to the standard medical care. A web-based, database-driven telemonitoring program for patients with IBD (TELE-IBD) previously examined in a randomized controlled trial was adopted in our model [14]. The IBD telemonitoring program used a mobile phone for patients, and a decision support server and website for providers. The patients received a series of texts weekly to grade their IBD symptoms and medication side effects. Self-assess body weight was also reported via text message. The telemonitoring program classified the patients, based on the weekly text response and predetermined criteria, to a disease activity zone. Clinical issues requiring immediate attention were directed to the provider’s office or on call service. Simultaneous action plans (including new prescriptions of medicine) and alerts were sent to the patients via telephone or text messages using the IBD telemonitoring program. Educational messages were also texted to the patients weekly. The relative change by healthcare avoidance in standard medical care during the pandemic was incorporated in both the SC and SC-TM groups. In the SC-TM group, the patient adherence to weekly self-assessment requirements was also considered.

### Clinical inputs

All model inputs are shown in **Table 1**. Literature search was conducted on MEDLINE between 2000 and 2022 using the keywords of “inflammatory bowel disease”, “ulcerative colitis”, “Crohn’s disease”, “COVID-19”, “avoidance”, “telemonitoring”, “compliance”, “adherence”, “hospitalization”, “surgery”, “mortality”, and “quality-adjusted life-year”. The clinical trial selection criteria were: (1) Reports written in English language; (2) adult patients with ulcerative colitis or Crohn’s disease; and (3) event (compliance, hospitalization, surgery, and/or mortality) rates and/or corresponding relative risk or odds ratios were reported. Meta-analyses and randomized controlled trials were the preferred studies. When multiple clinical trials were available for the same input parameter, the weighted average was used as the base-case value and the high/low values formed as the upper/lower limits for sensitivity analysis.

**Table 1:** Model inputs.

	Base-case value	Range for sensitivity analysis		Distribution	Reference
***Clinical inputs***					
Proportion of ulcerative colitis among IBD patients	0.568	0.483	0.654	Beta	20
Ulcerative colitis					
Hospitalization	0.186	0.158	0.213	Beta	25
Surgery in hospitalization	0.051	0.043	0.059	Beta	25
In-hospital mortality	0.003	0.003	0.003	Beta	26
Crohn’s disease					
Hospitalization	0.225	0.191	0.258	Beta	25
Surgery in hospitalization	0.331	0.282	0.381	Beta	25
In-hospital mortality	0.018	0.015	0.021	Beta	26
Percentage of patients with medical avoidance during pandemic	0.261	0.222	0.300	Beta	12
Relative risk of hospitalization associated with healthcare avoidance to standard care for IBD	1.156	1.050	1.329	Lognormal	27
Percentage of patients achieved telemonitoring compliance requirement	0.590	0.502	0.679	Beta	14, 29
Relative Risk of hospitalization associated with weekly IBD telemonitoring program	0.364	0.310	0.419	Lognormal	14
***Cost inputs***					
Ulcerative colitis (USD per patient year)					
Outpatients Visit	858	729	987	Gamma	25
Medications	3060	2607	3528	Gamma	25
Non-invasive diagnostic imaging	74	63	85	Gamma	25
Endoscopy	3,647	3,100	4,194	Gamma	25
Hospitalization	7,290	6,197	8,384	Gamma	25
Surgery	5,765	4,900	6,630	Gamma	25
In-hospital death	26,990	22,942	31,039	Gamma	25
Crohn’s disease (USD per patient year)					
Outpatients Visit	1,129	960	1,298	Gamma	25
Medications	2,894	2,460	3,328	Gamma	25
Non-invasive diagnostic imaging	485	412	558	Gamma	25
Endoscopy	3,328	2,829	3,827	Gamma	25
Hospitalization	9,389	7,981	10,797	Gamma	25
Surgery	5,534	4,704	6,364	Gamma	25
In-hospital death	44,596	37,907	51,285	Gamma	25
Relative reduction in cost of outpatient visits in patients with pandemic-related avoidance	26.1%	22.2%	30.0%	Lognormal	12
Telemonitoring program (USD per patient year)^a^	171	117	240	-	
IBD telemonitoring program annual maintenance (USD)	50	43	58	Gamma	31
Excessive electronic/telephone encounters mediated by IBD telemonitoring (per patient year)	4.760	4.046	5.474	Normal	14
Length of each IBD telemonitoring-mediated electronic/telephone encounter (hour)	0.250	0.213	0.288	Normal	31
Specialist medical staff cost (USD per hour)	101	86	116	Gamma	32
Relative increase of non-invasive diagnostic tests associated with IBD telemonitoring	1.565	1.331	1.800	Lognormal	14
***Utility inputs***					
Outpatients	0.830	0.789	0.872	Beta	33,34
Non-surgical Hospitalization	0.550	0.523	0.578	Beta	33,34
Surgical Hospitalization	0.400	0.380	0.420	Beta	33,34

IBD = Inflammatory bowel disease; USD1 = HKD7.8

^a^: Telemonitoring program (USD per patient year) = Annual maintenance fee+[Excessive encounters mediated by telemonitoring (per patient year)* Length of each encounter (hour)* Medical staff cost (USD per hour)

The prevalence of IBD patients with ulcerative colitis (56.8%) and Crohn’s disease (43.2%) was derived from the findings of a Hong Kong epidemiology study (n = 2,575) of a IBD registry [20]. The annual IBD-related hospitalization rate and event rate of major gastrointestinal surgery (including bowel resection, laparotomy, and stoma-related surgery) per hospitalization in patients with ulcerative colitis (18.6% and 5.1%) and Crohn’s disease (22.5% and 33.1%) were estimated from the findings of a retrospective 2-year follow-up study of Hong Kong IBD patients (n = 435) [25]. A 30-year retrospective cohort study (n = 1,467) in China reported the in-hospital mortality rates of hospitalized IBD patients to be 0.3% for ulcerative colitis and 1.8% for Crohn’s disease [26].

The percentage of medical avoidance among IBD patients (26.1%) was estimated from the results of a public survey (n = 765) on health service utilization in Hong Kong during the COVID-19 pandemic [12]. The hospitalization rate in IBD patients who avoided routine medical care during the pandemic were approximated by the relative risk of hospitalization associated with healthcare avoidance and the hospitalization rate prior to the pandemic. The relative risk of hospitalization (1.156) associated with healthcare avoidance to standard care was estimated using the primary care utilization data in the hospitalized and non-hospitalized IBD patients reported by a recent retrospective outcome study (n = 7,902 IBD patients) on the hospitalization rate and utilization of outpatient care in the past 12 months [27].

The relative risk of IBD-related hospitalization rate associated with weekly telemonitoring program (0.364) was approximated from the findings of a randomized controlled trial of the web-based telemonitoring program in 348 IBD patients [14]. The weekly IBD telemonitoring program required the patient compliance to be 80% or more in self-assessments [28–30] and the 59% of patients completed the required compliance level [14].

### Cost and utility inputs

The direct cost analysis was conducted from the perspective of Hong Kong healthcare provider. Costs were adjusted to the year 2021. The model time horizon was one year and discounting was therefore not applied to either cost or QALYs. The costs per patient year of IBD-related healthcare resources (speciality outpatient clinic visits, medications, diagnostic imaging and endoscopy, hospitalization and surgery) were retrieved from the findings of a retrospective 2-year follow-up study of the healthcare cost in 435 Hong Kong IBD patients. The cost of in-hospital death was approximated from the cost of IBD-related admission to the intensive care unit [25]. The cost per patient year of outpatient visits in IBD patients who experienced health service avoidance was estimated to reduce by 26.1%, based on the findings of the public survey (n = 765) on the change of health service utilization in Hong Kong during the COVID-19 pandemic [12].

The cost of telemonitoring program was estimated to include an annual maintenance fee, and costs of telemonitoring-mediated specialist consultation and non-invasive diagnostic tests. The annual maintenance fee (USD50; USD1 = HKD7.8) was approximated from the annual licence fee of a smartphone-based IBD program [31]. The telemonitoring-mediated medical staff cost was calculated by the excessive electronic/telephone encounters per patient year (4.76 encounters) mediated by the telemonitoring program, and the hourly wage of speciality physicians in Hong Kong [14, 32]. Each electronic/telephone encounter was charged for 15 minutes of medical staff cost based upon the clinical findings of telemonitoring-directed IBD care [31]. The cost per patient year of telemonitoring program was therefore estimated to be USD171. The relative increase of non-invasive diagnostic testings associated with telemonitoring (1.565) was adopted from the findings of the randomized controlled trial of the web-based telemonitoring program (n = 348) for IBD patients [14].

The expected QALYs gain were estimated by the health state-specific utility value. The health state preferences for general IBD population were previously measured by EuroQol-5 Dimension (EQ-5D), and the utility values generated by the EQ-5D measures were widely applied in cost-effectiveness analyses of IBD management [33–37]. The utility values (including outpatient care, non-surgical hospitalization and surgical hospitalization) in the present model were adopted from the same health-related quality of life studies on IBD [33, 34].

### Base-case analyses

All analyses were performed on TreeAge Pro Healthcare 2021 (TreeAge Software, Williamstown, MA, USA) and Microsoft Excel 2021 (Microsoft Corporation, Redmond, WA, USA). In analysis 1, the expected costs and QALYs of IBD standard care before (without healthcare avoidance) and during (with healthcare avoidance) the pandemic were compared. In analysis 2: The expected costs and QALYs of SC and SC-TM during the pandemic were compared. A treatment strategy was accepted as cost-effective when it gained higher QALYs at lower cost than another option. If a treatment strategy gained higher QALYs at higher cost than another alternative, incremental cost per QALY gained (ICER) of the more effective strategy was calculated:

ICER=ΔCost/ΔQALYs

The World Health Organization recommended that ICER less than 1× gross domestic product (GDP) per capita to be highly cost-effective and less than 3× GDP per capita to be cost-effective [38]. The GDP per capita of Hong Kong was USD 46,450 in 2020 [39] and it was adopted as the willingness-to-pay (WTP) threshold in the base-case analysis. A treatment alternative was preferred if (1) it was effective in gaining higher QALYs at lower cost, or (2) it was effective in gaining higher QALYs at an increased cost and the ICER was less than the WTP threshold.

### Sensitivity analyses

Sensitivity analyses were conducted to examine the robustness of model base-case results. In the deterministic sensitivity analysis, one-way analysis was performed to all model inputs (over the range of sensitivity analysis specified in **Table 1**) to identify the threshold value of influential parameters on the base-case results. The probabilistic sensitivity analysis was performed using Monte Carlo simulation. The direct cost and QALYs were recalculated 10,000 times by randomly drawing each of the model input from the parameter-specific distribution (**Table 1**). The incremental cost and incremental QALYs of SC-TM versus SC were presented in a scatterplot to examine the probability for SC-TM to be accepted as cost-effective.

## Results

### Analysis 1: IBD standard care with and without pandemic-related healthcare avoidance

The expected cost and QALYs are showed in **Table 2**. The IBD standard care (with pandemic-related avoidance) lost QALYs (by 0.0026 QALYs) and was more costly (by USD43), when compared with the outcomes of standard care without pandemic-related avoidance.

**Table 2:** Expected base-case results.

Strategy	Hospitalization	Mortality	Cost per patient year (USD)	Incremental costs per patient year (USD)	QALYs	Incremental QALYs
Analysis 1: Standard care						
Without pandemic-related avoidance	20.25%	0.20%	6902	-	0.7666	-
With pandemic-related avoidance	21.08%	0.21%	6945	43	0.7640	-0.0026
Analysis 2: During the pandemic						
Standard care	21.08%	0.21%	6945	-	0.7640	-
Standard care with telemonitoring	13.18%	0.13%	6146	-799	0.7888	0.0248

Hospitalization and mortality: Events per 100 patient-years

**Fig 2:**
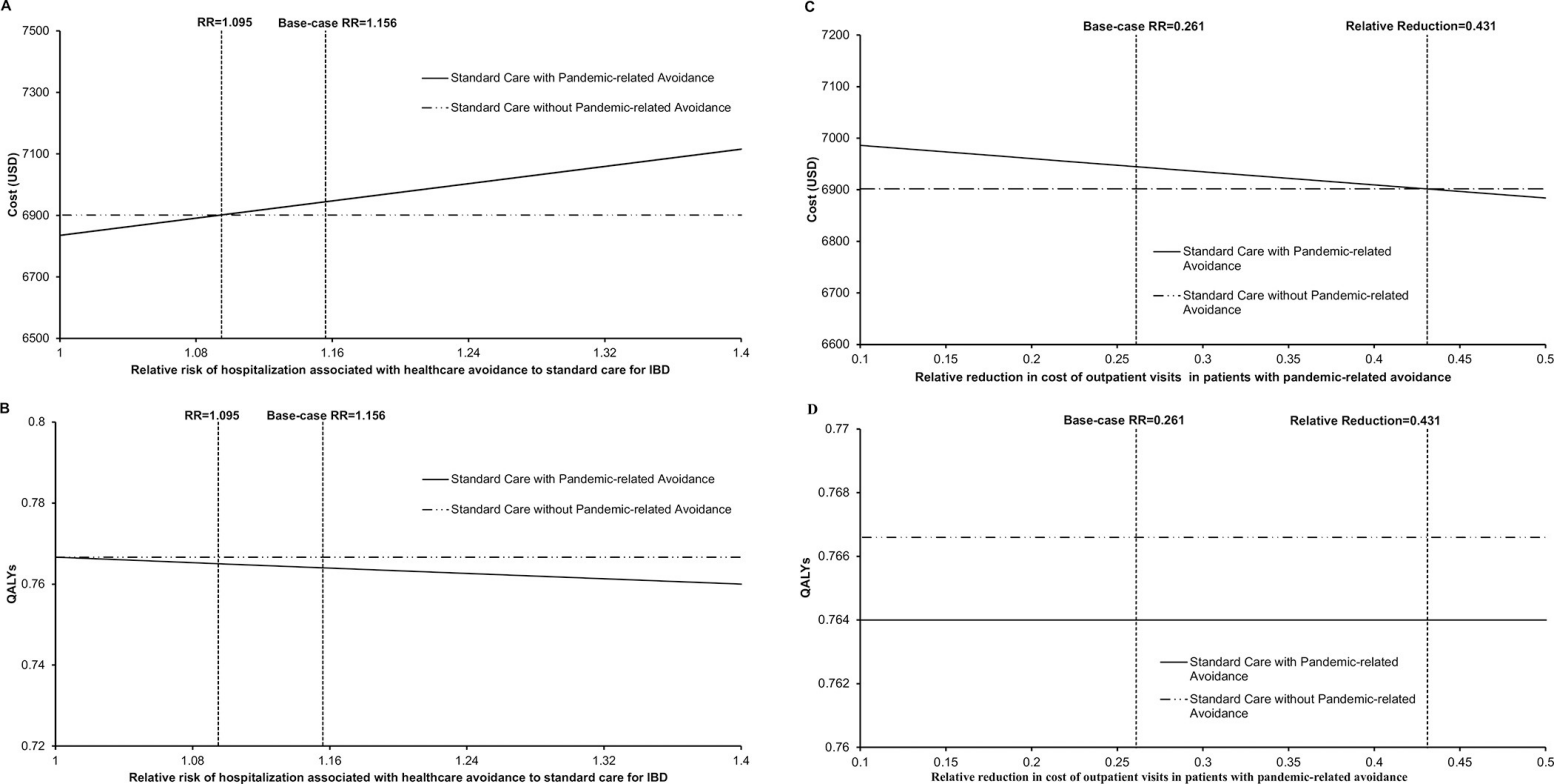
**A, B.** One-way sensitivity analysis on risk of hospitalization. **C, D.** One-way sensitivity analysis on outpatient cost reduction. IBD, inflammatory bowel disease; QALY, quality-adjusted life-year.

The one-way sensitivity analysis found two influential parameters: relative risk of hospitalization associated with healthcare avoidance to standard medical care (1.156; range 1.050–1.329) and relative reduction of outpatient cost in patients with pandemic-related avoidance (26.1%; range 22.2%-30.0%). The change of costs and QALYs against these two influential parameters are shown in **Fig 2A–2D**. The standard care with pandemic-related avoidance gained lower QALYs than the standard care without avoidance throughout the variation of these two variables (**Fig 2B and 2D**). When relative risk of hospitalization associated with healthcare avoidance was lower than 1.095, or when the relative reduction of outpatient cost in patients with avoidance was higher than 43.10%, the standard care with pandemic-related avoidance would become less costly than the standard care without avoidance (**Fig 2A and 2C**).

**Fig 3:**
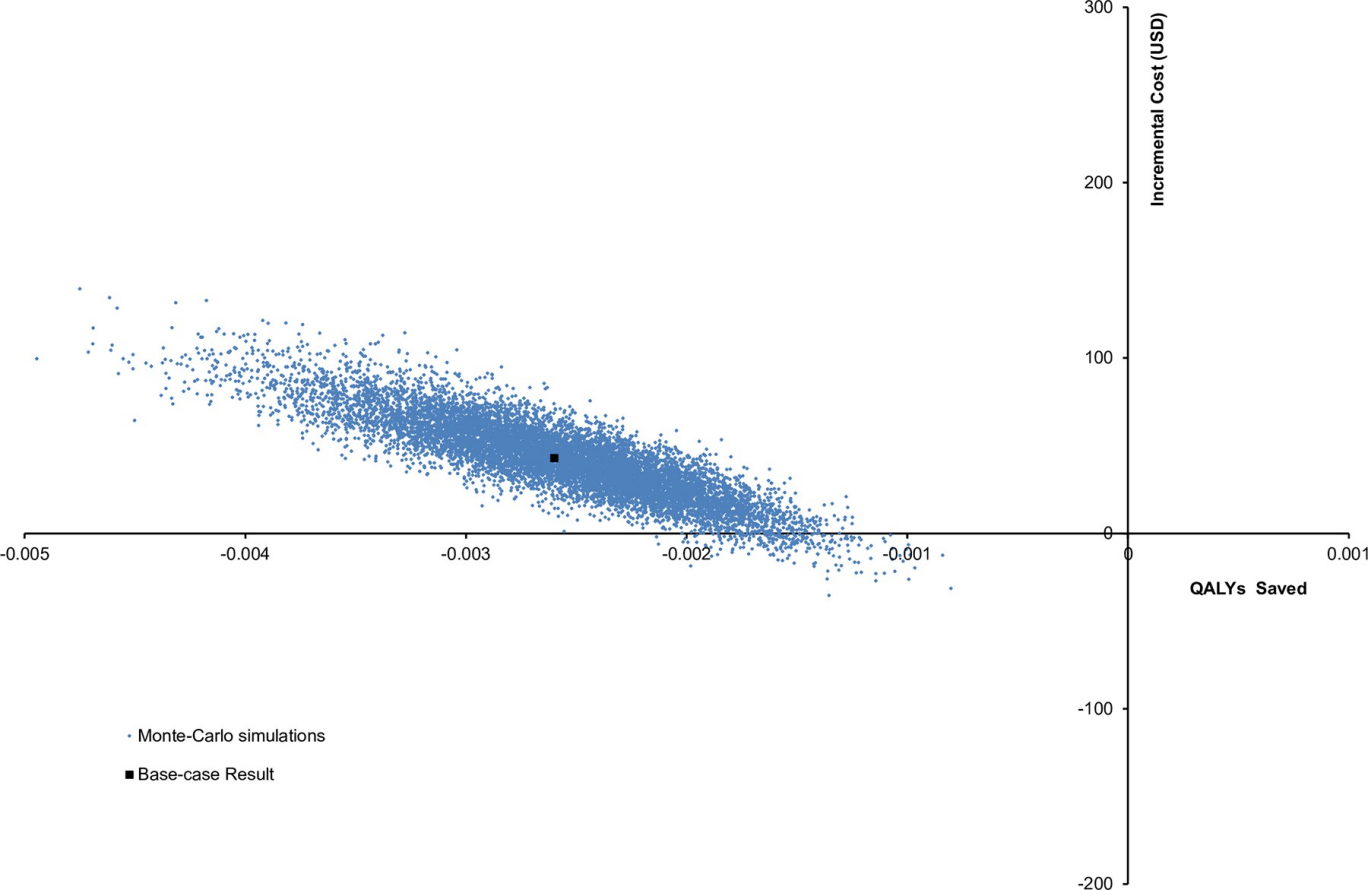
Scatterplot of change in cost against change in QALYs by standard care with pandemic-related healthcare avoidance. QALY: quality-adjusted life-year.

The probabilistic sensitivity analysis was performed by 10,000 Monte Carlo simulations. Comparing with IBD standard care without avoidance, the standard care with pandemic-related avoidance lost 0.0026 QALYs (95%CI 0.0025–0.0026; p<0.001) and increased the direct cost of care by USD42 (95%CI USD 42–43; p<0.001). **Fig 3** showed the scatterplot of the changes in cost and in QALYs by the standard care with pandemic-related healthcare avoidance. The standard care with pandemic-related avoidance lost QALYs in 100% of the 10,000 cohort simulations and incurred higher cost in 96.82% of the 10,000 cohort simulations.

### Analysis 2: Standard care with telemonitoring (SC-TM group) versus standard care alone (SC) during the pandemic

The base-case results are shown in **Table 2**. The SC-TM group gained higher QALYs (by 0.0248 QALYs) at lower total cost (cost-saving USD799) versus the SC group (with pandemic-related avoidance), and the SC-TM group was therefore the preferred cost-effective option.

**Fig 4:**
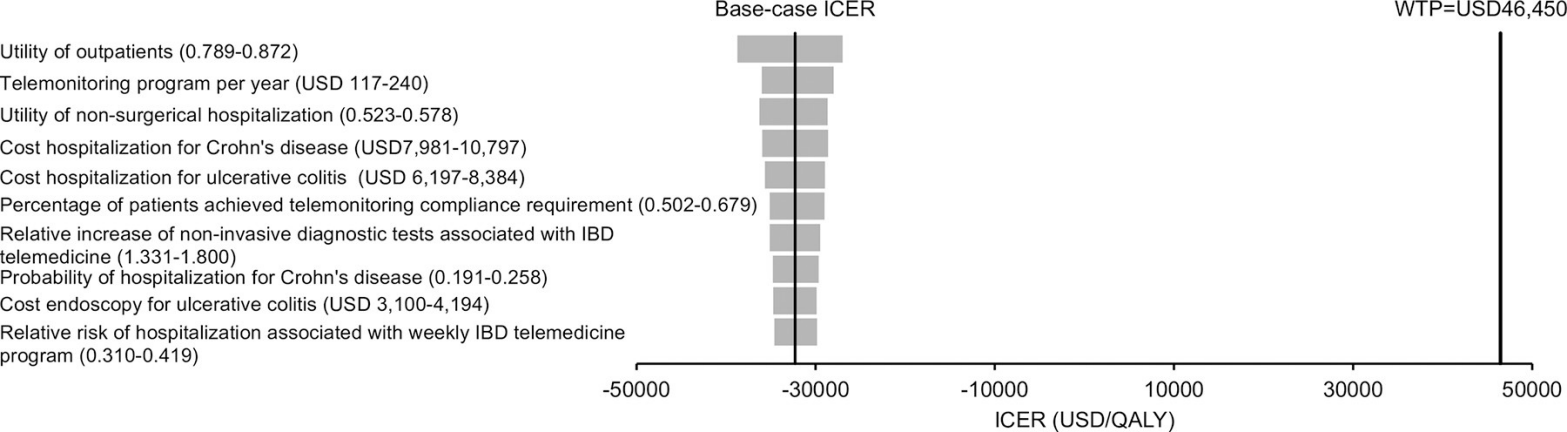
Tornado diagram of top ten influential factors identified on the ICER of SC-TM versus SC. QALY, quality-adjusted life-year; ICER, incremental cost‐effectiveness ratio; SC, standard care with pandemic-related avoidance; TM, telemonitoring; WTP, willingness-to-pay.

One-way sensitivity analysis showed that no threshold value was identified throughout the variation of all model inputs and the base-case results remained robust. The most influential parameters on the base-case ICER are showed in a Tornado diagram (**Fig 4)**.

Extended one-way sensitivity analyses were further conducted to explore the impact of the percentage of patients achieving telemonitoring compliance requirement and the cost of telemonitoring per patient-year on the cost-effectiveness of SC-TM. When the percentage of patients achieving compliance requirement for telemonitoring decreased from 0.590 (base-case value) to <0.133, or the telemonitoring cost increased from USD171 (base-case value) to >USD970 per patient-year, the total cost of SC-TM group would become higher than the total cost of SC group. When the percentage of patients achieving compliance requirement for telemonitoring further decreased to <0.067, or the telemonitoring cost increased to >USD2,119 per patient-year, the ICER of the SC-TM group would exceed the WTP threshold of 46,450 USD/QALY.

The percentage of patients with medical avoidance (base-case value: 26.1%; range: 22.2%-30.0%) was retrieved from a public survey on health service utilization in Hong Kong during the first peak of COVID-19 cases in early 2020 [12]. The medical avoidance rate might change in different stages of pandemic, and we further performed an extended one-way sensitivity analysis on the percentage of medical avoidance over a wide range of 0% to 50%. The extended one-way sensitivity analysis found that the SC-TM group remained to gain higher QALYs at lower costs when compared to the SC group.

**Fig 5:**
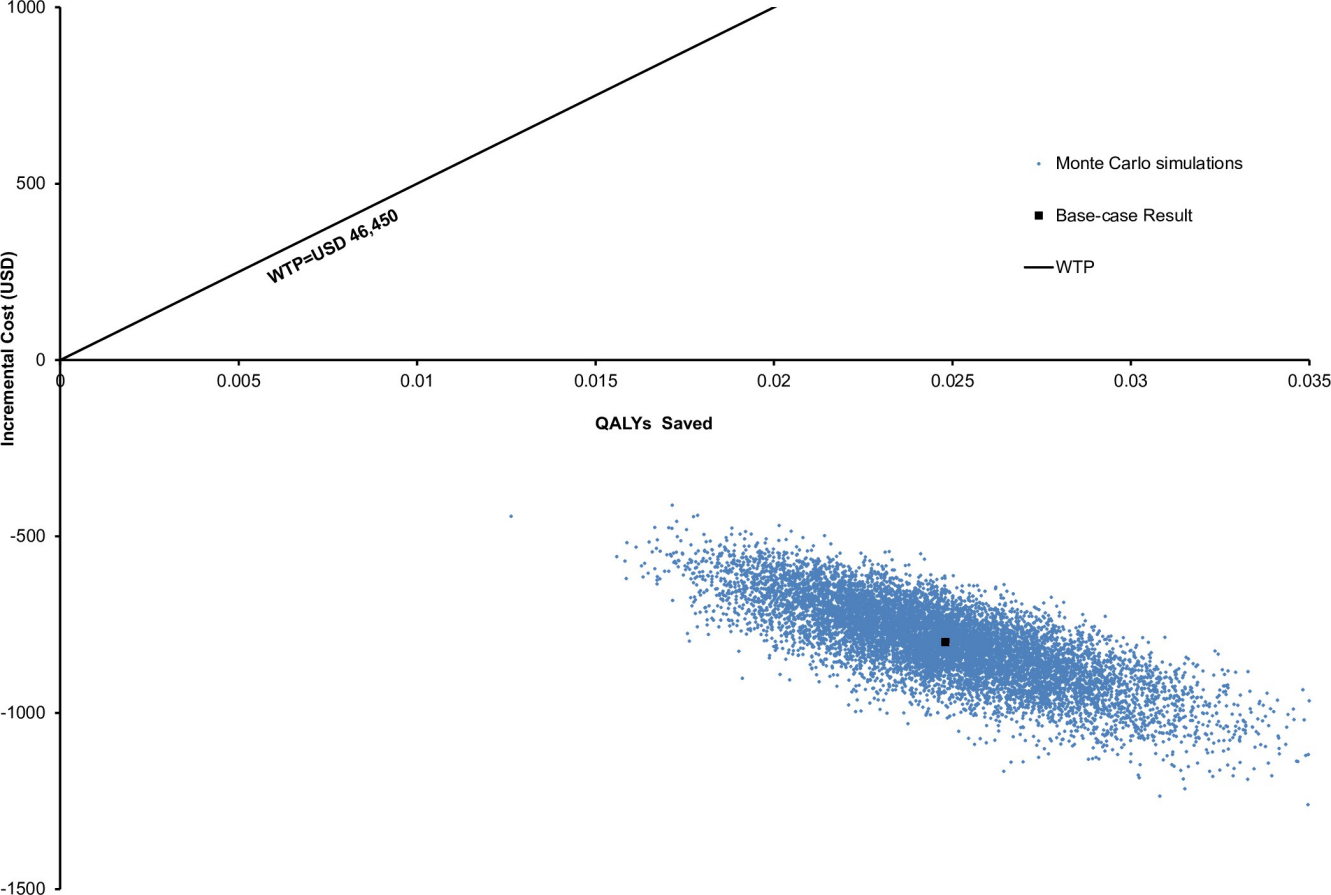
Scatterplot of incremental cost against QALYs saved by SC-TM versus SC from 10,000 Monte-Carlo simulations. QALY, quality-adjusted life-year; ICER, incremental cost‐effectiveness ratio; TM, telemonitoring; SC, standard care with pandemic-related avoidance; WTP, willingness-to-pay.

The costs and QALYs of SC-TM and SC groups were recalculated 10,000 times by Monte Carlo simulations. The incremental cost and incremental QALYs of SC-TM versus SC are shown in a scatterplot (**Fig 5)**. The SC-TM group gained higher QALYs at lower cost than the SC group in 100% of 10,000 cohort simulations. Compared with the SC group (with pandemic-related healthcare avoidance), the SC-TM group saved USD798 (95%CI USD796-800; p<0.001) and gained additional 0.0247 QALYs (95%CI 0.0247–0.0248; p<0.001).

## Discussion

This is the first outcome analysis to examine the impact of pandemic-related healthcare avoidance on health and economic outcomes of IBD patients, and evaluate the potential cost-effectiveness of adding telemonitoring to the standard care for IBD management during the COVID-19 pandemic. Compared with IBD standard care without pandemic-related avoidance, the care under COVID-19 (with pandemic-related avoidance) lost 0.0026 QALYs and increased cost by USD 43 per patient over a one-year period. The COVID-19 pandemic had a ripple effect on the IBD management: The pandemic inflicted health service avoidance (due to fear of acquiring COVID-19 in healthcare facilities), causing IBD-related routine care utilization to reduce. Due to the impact of healthcare avoidance to standard medical care, the risk of hospitalization of IBD patients was therefore elevated and subsequently increased overall cost and reduced QALYs.

One-way sensitivity analysis found the QALYs loss with pandemic-related avoidance was robust to variation of all model inputs. The most influential parameters on the increased cost during the pandemic are the relative risk of hospitalization and relative reduction in outpatient cost associated with healthcare avoidance. Probabilistic sensitivity analysis further supported the base-case findings that the standard care with healthcare avoidance lost QALYs (at 100% of time) at increased cost (at >96% of time).

Adding telemonitoring to the standard care (SC-TM) gained higher QALYs (by 0.0248 QALYs) and saved cost by USD 799 when compared with standard care alone (SC) with pandemic-related avoidance over a one-year period. The increment QALY gain and cost saving were generated by the reduction in hospitalization (and subsequently reduced number of in-hospital deaths). Results from one-way sensitivity analysis indicated that the base-case findings were robust and no influential parameters was identified. The cost of telemonitoring program per year and the percentage of patient achieving required compliance level to telemonitoring are modifiable variables in the model. We further conducted the extended one-way sensitivity analysis, and found the lower threshold of the percentage of patients achieving required compliance (>0.067) and the upper threshold of the telemonitoring cost per patient-year (<USD 2,119) for SC-TM to be accepted as cost-effective (ICER<WTP). The robustness of base-case findings was also supported by probabilistic sensitivity analysis that 100% of Monte Carlo simulations found the SC-TM to be the preferred strategy.

Findings of the present study were consistent with the previous cost-effectiveness analyses on telemonitoring programs for IBD. The myIBDcoach trial-based cost-effectiveness analysis in the Netherlands found that telemonitoring-directed strategy saved USD612 per patient in a year and the telemonitoring strategy was cost-effective in 83% of the time [31]. The TECCU trial-based cost-effectiveness analysis in Spain reported that telemonitoring strategy saved USD2,463 per patient in remission in a 24-weeks period and telemonitoring was accepted to be cost-effective in 84% of time [40]. It is plausible that the difference in cost-saving between the studies was a result of the choices of perspectives adopted in different countries for cost estimation. Both previous cost-effectiveness analyses in the Netherlands and Spain adopted a societal perspective and took direct and indirect costs into consideration [31, 40], whilst the present study was performed from the perspective public healthcare provider in Hong Kong on direct medical cost analysis.

The present study was limited by a short time horizon (one-year) applied in the decision-analytic model. The model was also limited by simplifying the IBD treatment outcomes in two key statuses: Outpatient care and IBD-related hospitalization. The morbidity of IBD-related hospitalization caused by exacerbation of IBD symptoms or occurrence of IBD-related complications usually results in high direct medical cost and reduction in health-related quality of life, and the total management cost and QALY of IBD are therefore highly sensitive to the change of IBD-related hospitalization rate. IBD-related hospitalization is a strong indicator of the performance of IBD outpatient management in terms of treatment cost and QALYs gained. Nevertheless, the simplified model did not fully represent the complexity of settings and treatments available for IBD patients, and could weaken the validity and reliability of the results. For instance, the patients who avoided standard outpatient care (amid the pandemic) might still stay in outpatient care (without hospitalization), yet experience worsened IBD symptoms. The loss of QALYs in patients who experienced pandemic-related avoidance to healthcare service might therefore be underestimated. The age-specific acceptance to mobile technology was not considered in the model. The smartphone penetration rate was 91.5% in Hong Kong, yet only 66.7% in the subgroup aged 65 years and over [41]. The benefit of telemonitoring might therefore be influenced by acceptance rate among the elderly subgroup of IBD patients. Future study on acceptance of telemonitoring across age groups is highly warranted. The key clinical inputs of the telemonitoring were derived from the findings reported in one overseas randomized controlled trial, and might affect the generalizability of model results to a larger IBD population in Hong Kong. Rigorous sensitivity analyses were therefore performed to examine the impact of uncertainty of all variables on the robustness of model outcomes, and no threshold value of influential model input was identified.

The COVID-19 pandemic has challenged the healthcare systems worldwide to maintain routine disease management. In Hong Kong, COVID-19 patients have been the priority of the healthcare system. As a result, the non-urgent medical services are unavoidably deferred. Nonetheless, patients also concern about the risk of acquiring COVID-19 at healthcare facilities and therefore avoid medical services. The present findings established the loss in both QALYs and costs associated with the pandemic-related avoidance in standard outpatient care, and demonstrated the potential cost-saving and QALYs gained by adding a telemonitoring program to the current care for IBD patients. Despite the high internet accessibility, the development of telemonitoring in Hong Kong is still in its infancy. The present findings supported the development of telemonitoring (suggested by the public officials’ of Hong Kong) as part of the contingency plan to provide non-urgent healthcare service during the peak of the pandemic [42, 43]. With the paucity of research on telemonitoring for long-term IBD management, both clinical study and cost-effectiveness analysis of telemonitoring-mediated long-term IBD management are highly warranted to provide evidence to inform clinicians, patients and policy makers.

## Conclusion

The standard outpatient care for IBD patients during the pandemic with healthcare avoidance (versus care prior to the pandemic) appears to worsen the treatment outcomes at higher cost and lowered QALYs. The addition of telemonitoring to the standard care during the pandemic seems to gain higher QALYs and save cost, and therefore a potential cost-effective strategy for IBD management during the pandemic.
